# Bioactive Potential of 3D-Printed Oleo-Gum-Resin Disks:* B. papyrifera*,* C. myrrha*, and* S. benzoin* Loading Nanooxides—TiO_2_, P25, Cu_2_O, and MoO_3_

**DOI:** 10.1155/2017/6398167

**Published:** 2017-07-25

**Authors:** Diogo José Horst, Sergio Mazurek Tebcherani, Evaldo Toniolo Kubaski, Rogério de Almeida Vieira

**Affiliations:** ^1^Federal University of Technology-Paraná, 84016-210 Ponta Grossa, PR, Brazil; ^2^Department of Production Engineering, Federal University of Technology-Paraná, 84016-210 Ponta Grossa, PR, Brazil; ^3^Department of Materials Science, State University of Ponta Grossa, 84030-900 Ponta Grossa, PR, Brazil; ^4^Department of Earth and Exact Sciences, Federal University of São Paulo, 89972-270 São Paulo, SP, Brazil

## Abstract

This experimental study investigates the bioactive potential of filaments produced via hot melt extrusion (HME) and intended for fused deposition modeling (FDM) 3D printing purposes. The oleo-gum-resins from benzoin, myrrha, and olibanum in pure state and also charged with 10% of metal oxide nanoparticles, TiO_2_, P25, Cu_2_O, and MoO_3_, were characterized by ultraviolet-visible (UV-Vis) and Fourier transform infrared (FTIR) spectroscopy, energy-dispersive X-ray microanalysis (EDXMA), scanning electron microscopy (SEM), and differential scanning calorimetry (DSC). Disks were 3D-printed into model geometries (10 × 5 mm) and the disk-diffusion methodology was used for the evaluation of antimicrobial and antifungal activity of materials in study against the clinical isolates:* Staphylococcus aureus, Pseudomonas aeruginosa, Escherichia coli*, and* Candida albicans*. Due to their intrinsic properties, disks containing resins in pure state mostly prevent surface-associated growth; meanwhile, disks loaded with 10% oxides prevent planktonic growth of microorganisms in the susceptibility assay. The microscopy analysis showed that part of nanoparticles was encapsulated by the biopolymeric matrix of resins, in most cases remaining disorderly dispersed over the surface of resins. Thermal analysis shows that plant resins have peculiar characteristics, with a thermal behavior similar to commercial available semicrystalline polymers, although their structure consists of a mix of organic compounds.

## 1. Introduction

Infections caused by pathogenic microorganisms are of great concern in many fields [1]. Hospital-acquired infections are one of the major problems increasing mortality and morbidity. Microbial contamination may occur from various sources and invasive interventions [2].* Staphylococcus aureus* is a Gram-positive major human pathogen that causes skin and soft-tissue infections, life-threatening infections such as pneumonia and sepsis, and toxinoses including toxic shock syndrome [3].* Pseudomonas aeruginosa* is a Gram-negative bacillus that is rapidly becoming one of the major causes of opportunistic and nosocomial infections which have become a worldwide problem [4].* Escherichia coli* is a Gram-negative pathogen which has been regarded as an important indicator bacterium; it causes severe diseases, such as hemolytic uremic syndrome (HUS), hemorrhagic colitis [5], and thrombotic thrombocytopenic purpura which can be fatal in some cases [6, 7].* Candida albicans* is renowned as the leading fungal pathogen of oral candidosis, which manifests in a variety of clinical guises ranging from common denture associated infections in otherwise healthy individuals to systemic infections in human immunodeficiency virus disease [8, 9].

Although bacteria have shown the ability to acquire resistance to many antibiotics, however, in nature, there are several examples of antibiotics to which resistance has not yet developed [10, 11]. The history of medicine and pharmacy is well known for using plant oleo-gum-resins and extracts in curing diseases; these are known to have analgesic, antioxidant, antifungal, antiseptic, antibacterial, astringent, sedative, and stimulant therapeutic properties, among others [12–21]. The bioactivity of oils and extracts obtained species* Commiphora myrrha, Styrax benzoin*, and* Boswellia papyrifera* has been investigated by several researches [22–31]; these aromatic resins basically consist of monoterpenes (C10H16), triterpenes (C30H48), and sesquiterpenes (C15H24) with unique combinations, besides benzoic, myrrholic, and boswellic acids, respectively [32–37]; the demonstration of the presence of secondary metabolites in medicinal plants oils, extracts, and resins provides a scientific validation for the popular use of these plants [38–42].

Besides that, a greener approach for the biosynthesis of colloidal metal nanoparticles and dispersion/encapsulation of drugs using natural oleo-gum-resins has been suggested as being effective and more environmental friendly [43–49]. Moreover, the development of multifunctional nanocomposite materials with enhanced mechanical and antimicrobial properties has been studied [50–52]. Indeed, nanoparticles (NPs) are widely used in the field of healthcare, presenting numerous advantages in medical and biotechnological applications [53, 54] and increasingly attracting researchers due to their unique properties, such as submicrometer size (1–100 nm), large surface-to-volume ratio, and advanced reactivity [55, 56].

It is imperative to improve the applicability of 3D printing for pharmaceutical purposes by searching novel materials. The investigation of different physicochemical properties and adequate processing parameters of such materials is important for successful additive manufacturing of personalized geometries [57]. In the last years, the use of 3D printing for the development of drug delivery systems, medical devices, bone tissue engineering, and antimicrobial materials has shown promising results with a large possibility of applications [58–61]. Beyond that, the use of hot melt extrusion (HME) in the fabrication of novel antimicrobial filaments for pharmaceutical application has steadily increased [62–67]. Nature can combine brittle minerals and organic molecules into hybrid composites that are highly organized to achieve exceptional properties [68, 69]; organic-inorganic hybrid nanostructures and materials on their basis are promising class of multifunctional advanced materials [70, 71]. Within this context, the purpose of this study was to evaluate the potential of hybrid engineered materials intended for fused deposition modeling (FDM) 3D printing, testing its bioactivity against clinical pathogenic organisms including Gram-positive, Gram-negative bacteria, and fungus.

## 2. Materials and Methods

### 2.1. Materials and Reagents

Oleo-gum-resins from benzoin* (Styrax benzoin)* harvested in Singapore, olibanum* (Boswellia papyrifera)* originally from Ethiopia, and myrrh* (Commiphora myrrha)* from Somalia were purchased from Mountain Rose Herbs (Eugene, Oregon, USA). Titanium dioxide (TiO_2_), anatase (P25), molybdenum trioxide (MoO_3_), and copper I (Cu_2_O) oxide were purchased from Plasmachem (GmbH, Germany); the nanoparticles have sizes between 10 nm to 1 nm. Prior to testing, all materials were sterilized using UV radiations. Clinical isolates of* Staphylococcus aureus* ATCC 6538*; Pseudomonas aeruginosa* ATCC 9027;* Escherichia coli* ATCC 8739; and* Candida albicans *ATCC 2091 were purchased from the American Type Culture Collection (ATCC, Manassas, VA, USA). Mueller-Hinton agar and Sabouraud dextrose agar were purchased from Kasvi (Curitiba, PR, Brazil).

### 2.2. Preparation of Materials

Resins in powder form in pure state and also loaded with 10% (w.t) oxides were added to a hot melt desktop screw extruder (Filastruder, GA, USA) forming printable filaments measuring 1.75 mm diameter; the extrusion was performed at temperatures within 70–85°C and the materials were cooled at ambient conditions; the extrusion speed was maintained at 20 rpm. In sequence, disks (10 × 5 mm) were manufactured using a FDM 3D printer (Prusa Mendel–I3, USA). The printing temperature was maintained at 80°C and the heating of table was maintained at 60°C; the printing feeding speed was maintained at 10 mm/min; the output measure of the hot end is 0.4 mm.

### 2.3. Susceptibility Assay In Vitro

Mueller-Hinton agar (MHA) was used to determine the antimicrobial activity of materials in study against bacteria and Sabouraud dextrose agar (SDA) was prepared for fungi; the agar media were prepared by following manufacturer instructions. The plates were autoclaved, in sequence; holes were made for insertion of sampling disks. Using a sterile transfer loop, suspensions containing 5 × 10^6^ CFU/mL^−1^ were inoculated in the agar media. Then, the plates were incubated at bacteriological greenhouse during 48 hours at 34°C, with growth checks performed at every 6 hours. The bioactivity of materials was evaluated by comparing the inhibition zone (diameter in mm) and also number of colony forming units (CFU) in relation to the control plates with positive growth. The semiquantitative K-B disk-diffusion method was used to determine the antimicrobial and antifungal activity of materials [72, 73]. To obtain a more accurate counting of the viable cells, the software ImageJ with the plugin automated colony counting was used [74–76].

### 2.4. Scanning Electron Microscopy (SEM) and Energy-Dispersive X-Ray Microanalysis (EDXMA)

Scanning electron microscopy (SEM) analysis was performed using equipment Jeol (JSM-6610LV). Samples of resins in pure state and resins loading metal oxides were placed on a carbon tape and subjected to a vacuum and electron beam. The structural identification of the samples was performed by X-rays diffraction measurements; the equipment used was a Bruker diffractometer (D8 Advance) equipped with a lynx-eye detector. We used a copper X-ray generator tube with radiation Kalfa 1 = 1.5406 A. The power was adjusted to 1600 W (40 kV and 40 mA) for evaluating the signals diffracted in the region between 20° and 120° 2 theta in step 0.025°/s. Samples were prepared in order to avoid any preferential orientation of hkl planes in a standard circular sample holder with a diameter of approximately 2.5 cm.

### 2.5. UV-Visible (UV-Vis) Absorption and Fourier Transform Infrared (FTIR) Spectroscopy

Spectroscopy analysis in the ultraviolet-visible region (UV-Vis) was carried out utilizing Shimadzu equipment (UV-1800) calibrated at a bandwidth of 1 nm; the wavelength range was maintained from 300 to 900 nm. The Fourier transformed infrared (FTIR) spectroscopy measurements were performed using a Shimadzu Spectrometer Prestige 21 (Shimadzu Corporation, Koyoto, Japan) with a resolution of 2 cm^−1^. The measurements were carried out on KBr pellets which have transparency in the infrared region 400–4000 cm^−1^. In this context, the powder samples resins and resins oxides (1 mg) were ground with KBr (300 mg, spectroscopic, high purity). To form the tablets, the mixture was placed in a hydraulic press applying approximately 10 tones, while the air was extracted by a mechanical pump. The FTIR spectra of the samples were recorded at ambient temperature and spectral band between 4000 and 300 cm^−1^.

### 2.6. Differential Scanning Calorimetry (DSC)

In the calorimetric analysis, samples were weighed (3.0 ± 0.5 mg) and hermetically sealed in aluminum crucibles being placed in a Shimadzu calorimeter model DSC-60 under an atmosphere of nitrogen, flow 50 ml min^−1^; the heating ratio was maintained from 20°C min^−1^ to 550°C. The heating rate was maintained at 10°/min and the nitrogen flow was 100 ml/min. The equipment was calibrated for temperature with indium standard (156.6 ± 0.3°C) through their melting peak. The enthalpy and heat flow were calibrated using the heat of fusion of indium (28.59 J/g ± 0.30) using the same conditions as the samples. The correction factor was calculated in accordance with the procedures and specifications from Shimadzu.

### 2.7. Statistical Analysis

For data treatment of the susceptibility assay in vitro, the Experimental Design in Contextualized Blocks was used [77, 78]. In this research, the blocks are the 16 samples (raw data) and the treatments are 15 types of material + control plates, therefore totalizing 16 essays; the assay was carried out in quadruplicate for tested microorganisms; for data treatment, the statistical software SASM-Agri-8.1 was used [79].

### 2.8. List of Abbreviations

In text, the resins in pure state were identified as follows:* Styrax benzoin* (B pure),* Commiphora myrrha* (M pure), and* Boswellia papyrifera* (P pure). The oxides were identified as titanium dioxide (TiO_2_); anatase oxide (P25); copper I (Cu_2_O); and molybdenum trioxide (MoO_3_), respectively.

## 3. Results and Discussion

### 3.1. Susceptibility Assay In Vitro

**Figure 1:**
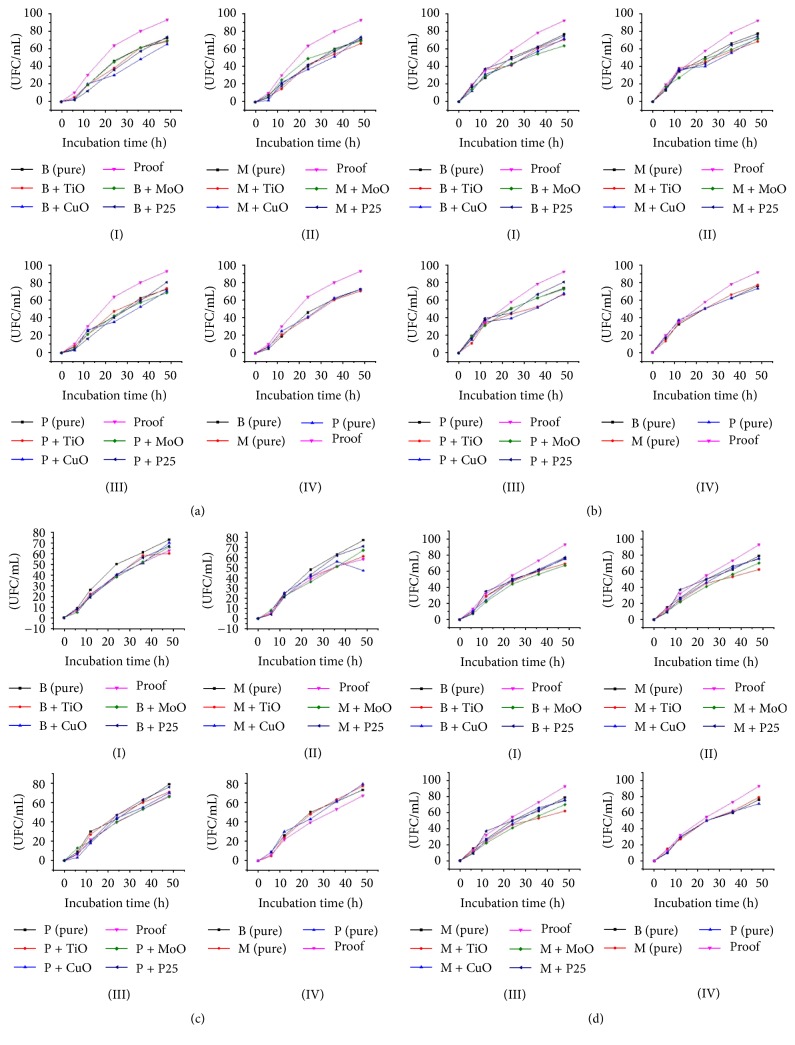
Antibiogram of clinical isolates: (a) refers to* C. albicans*; (b) refers to* E. coli*; (c) refers to* S. aureus*; and (d) refers to* P. aeruginosa *microorganisms.

According to Figure 1(a) during the initial period of 6 hours, the growth of* C. albicans* was inhibited by materials B (pure), B + TiO_2_, B + MoO_3_, B + Cu_2_O, B + P25, P + MoO_3_, P + Cu_2_O, P + P25, M + MoO_3_, M + Cu_2_O, B + MoO_3_, B + Cu_2_O, B + P25, P + Cu_2_O, and M + Cu_2_O (*p* = 0.05).

In 12-hour testing period, its growth of was limited by resin B (pure), B + TiO_2_, B + MoO_3_, B + Cu_2_O, B + P25, P + TiO_2_, P + MoO_3_, P + P25, M pure, M + TiO_2_, M + P25, B + P25, P + P25, and M + TiO_2_. During 24-hour period, its growth was hampered by all materials, with one exception M + MoO_3_; at this period, the most efficient materials were B + Cu_2_O, B + P25, and P + Cu_2_O. At 36 hours, B + Cu_2_O, P + Cu_2_O, M + Ti_2_O, and M + Cu_2_O were the most efficient materials. In the test period of 48 hours, its growth was restrained by all materials with one exception in P + P25 and the most efficient materials were B (pure), B + TiO_2_, B + MoO_3_, B + Cu_2_O, P + MoO_3_, P + Cu_2_O, M (pure), M + TiO_2_, and M + MoO3. In short, all materials tested (numerically) impede the proliferation of* C. albicans* (*p* = 5%), highlighting B + TiO_2_, B + MoO_3_, B + Cu_2_O, P + MoO_3_, P + Cu_2_O, M (pure), and M + MoO_3_ which were the most efficient bioactive materials during the 48-hour assay.

According to Figure 1(b), during initial 6-hour testing period, the most effective materials were B + Cu_2_O, P + TiO_2_, M (pure), M + Cu_2_O, and M + P25. Already during 12-hour testing period, the materials B (pure), B + MoO_3_, P + MoO_3_, and M + MoO_3_ were the most effective. During 24-hour assay, the materials B + TiO_2_, B + MoO_3_, B + Cu_2_O, P + TiO_2_, P + Cu_2_O, M + TiO_2_, M + Cu_2_O, and M + P25 were the most efficient and, during 36-hour period, the materials B + MoO_3_, P + TiO_2_, P + Cu_2_O, M + TiO_2_, and M + Cu_2_O were the most effective. At the final period of 48-hour assay, all materials showed significant effectiveness in relation to the control plate, with only one exception P + P25 (*p* = 5%). In sum, all materials under study (numerically) prevent the proliferation of* E. coli*, highlighting B + MoO_3_, P + Cu_2_O, P + TiO_2_, and M + TiO_2_, which stood out as the most bioactive materials.

In relation to Figure 1(c), during the initial period of 6 hours, among the materials that inhibited the growth of this bacterium, B + MoO_3_, B + Cu_2_O, P + Cu_2_O, and M + P25 stood out. However, in the period of 12 hours, all materials tested showed no significant difference concerning the control plates, being considered ineffective during this period of assay. In 24-hour period, the materials B + Cu_2_O, B + TiO_2_, B + MoO_3_, B + Cu_2_O, B + P25, P + MoO_3_, M + TiO_2_, M + MoO_3_, and M + Cu_2_O were the most effective in detaining its growth. In the period of 36 hours, B + MoO_3_, B + Cu_2_O, P + Cu_2_O, M + TiO_2_, and M + Cu_2_O were the most efficient. At the final period of 48 hours, the materials B + MoO_3_, B + P25, P + MoO_3_, M + TiO_2_, and M + MoO_3_ were the most effective in inhibiting its growth (*p* = 5%). In short, all materials (numerically) were effective against the bacteria in question, highlighting B + MoO_3_, P + MoO_3_, and M + TiO_2_ and presenting the higher inhibition rates.

According to Figure 1(d) during the initial period of 6 hours, in all cases, there was no significant difference between the materials and the control plate. However, during 12-hour period, significant differences were noted, highlighting B + MoO_3_ and M + MoO_3_ which were the most effective in restraining its growth. In the period of 24 hours, in most cases, there was no significant difference between the materials and control plates, with just one exception M + MoO_3_. In the time interval of 36 hours, in most cases, there were significant differences; it is noteworthy that B + MoO_3_, M + TiO_2_ and M + MoO_3_ were the most bioactive. In the period of 48 hours, in all cases, there were no significant differences between the materials and control plate, with just one exception M + TiO_2_, highlighting that B + MoO_3_, M + MoO_3_, and M + TiO_2_ were the most efficient biocides in inhibiting the proliferation of* S. aureus*.

Table 1 shows the results of the antibacterial and antifungal activity of materials against the tested pathogenic microorganisms; the results are exposed as the mean values obtained after 48 hours of assay.

The results from Table 1 show that the bioactivity of plant resins in pure state was effective and the addition of 10% w.t of oxides nanoparticles increased the efficiency of materials, as expected. Regarding the resin of* C. myrrha*, the antibiogram of bacteria and fungus under test corroborates the results obtained by Omer et al. [80] and in Alhussaini et al. [81]; concerning the resin of* B. papyrifera*, the results present in this study are in accordance with Camarda et al. [25], Abdallah et al. [82], Abdalah and Khalid [83], and de Rapper et al. [84]; and in relation to the resin of* S. benzoin* the results showed here are in accordance with the findings previously obtained by Dahni et al. [85] and De Rapper et al. [86].

### 3.2. SEM and EDXMA

**Figure 2:**
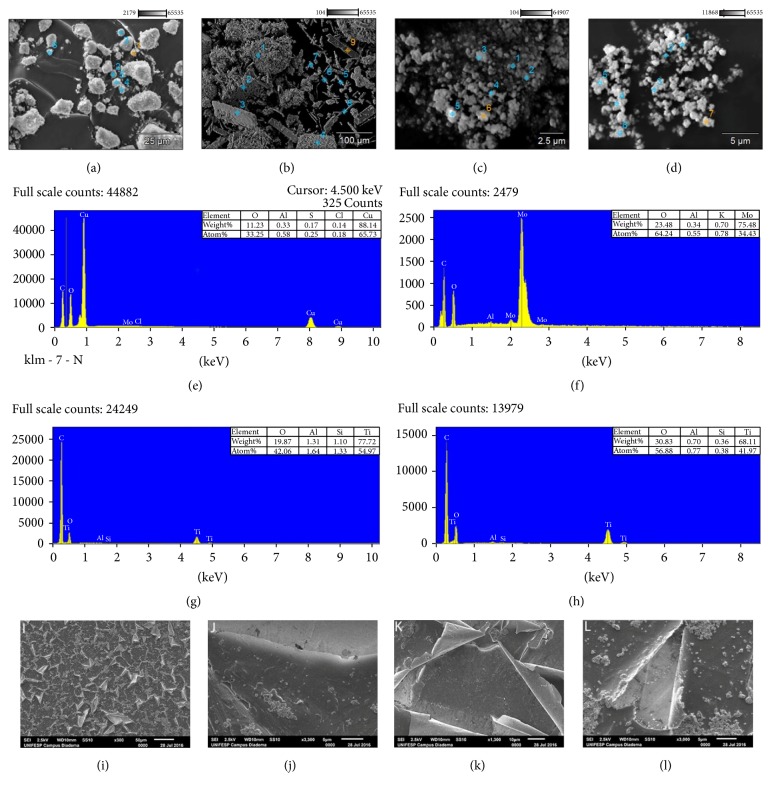
EDXMA analysis of metal oxides: Cu_2_O (a); MoO_3_ (b); P25 (c); TiO_2_ (d); Cu_2_O (e); MoO_3_ (f); P25 (g); TiO_2_ (h). SEM analysis of materials: B + P25 (i); M + TiO_2_ (j); P + Cu_2_O (k); B + MoO_3_ (l).

Figure 2 shows the results of scanning electronic microscopy and energy-dispersive X-ray microanalysis.

According to Figure 2, the SEM analysis showed that part of oxides nanoparticles was encapsulated by the matrix of resins, and part remained heterogeneously dispersed over the surface of resins. Therefore, better ways of addition of oxides need to be studied, aiming for homogeneous nanostructure of the materials in the best way possible. One option is to increase the shearing forces during extrusion or premixing the content could improve the dispersant size and homogeneity in the dispersion by fractioning large agglomerates [87]. Another approach could be to feed the nanoparticles in suspension into the extrusion line [88]. During the printing of sample disks, an incomplete dispersion of the colloidal oxides in the disks was observed in the form of microsized domains of agglomerated nanoparticles; such agglomeration has previously been reported as a general problem for ceramic nanoparticles dispersed in polymers [89].

**Figure 3:**
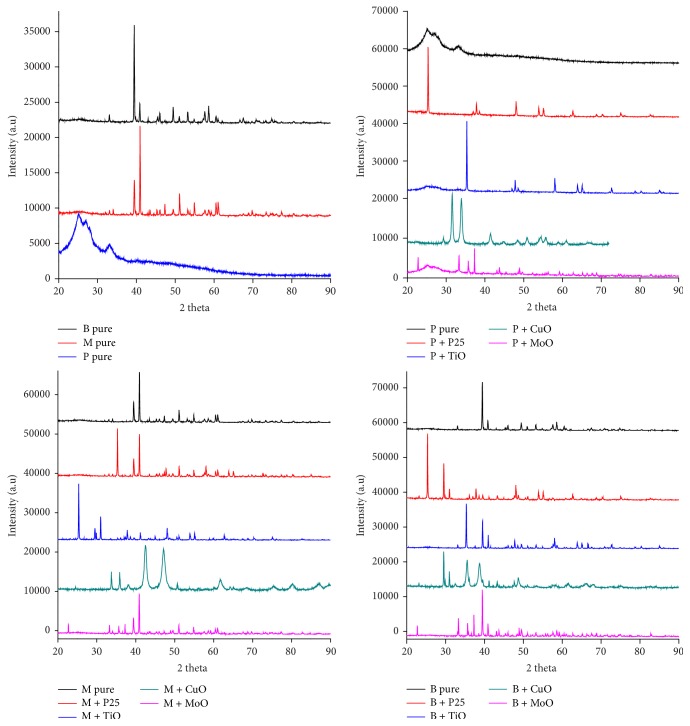
X-ray diffractograms of samples in pure state and also doped with metal oxides nanoparticles.

As exposed in Figure 3, the EDXMA analysis revealed information about the crystalline structure of samples.

According to Figure 3, the peaks identified at 2*θ* = 15° confirm the crystalline structure of the* B. papyrifera* [90]; the peaks found at 2*θ* = 35° and 45° confirm the molecular structure of* C. myrrha* [91]; the peaks at 2*θ* = 35° and 40° match with the chemical structure of* S. benzoin* [92]. No secondary phase was found samples, so it was possible to index the monoclinic phase of titanium oxide (PDF number 65-5714) and P25 anatase + rutile (PDF number 21-1272 and 21-1276, respectively). Those peaks at scattering angles of 25.26°, 36.94°, 48.05°, 53.89°, 55.06°, and 62.681° correspond to the reflections from the (101), (004), (200), (105), (211), and (204) crystalline planes of anatase (P25) and TiO_2_ oxides [93]; it was possible to index the monoclinic phase of copper I (PDF number 89-5898) and molybdenum oxide (PDF number 13-345); the diffraction peak found at 2*θ* = 26° found in sample B + P25 is attributed to the hexagonal structure of graphite (002) [94]; the diffraction peaks found at 2*θ* = 29,6°, 36,7°, and 42,5° are attributed to Cu_2_O crystalline planes (110), (200), and (220), respectively [95]. Lastly, the diffraction peaks at 2*θ* around 23.20°, 25.51°, 27.18°, 33.66°; 38.76°; 52.64°; 58.79°; and 67.28° correspond to (110), (040), (021), (111), (131), (211), (081), and (261); XRD standard data planes indexed to pure monocyclic and orthorhombic structure of MoO_3_ [96].

### 3.3. UV-Vis and FTIR Spectroscopy

**Figure 4:**
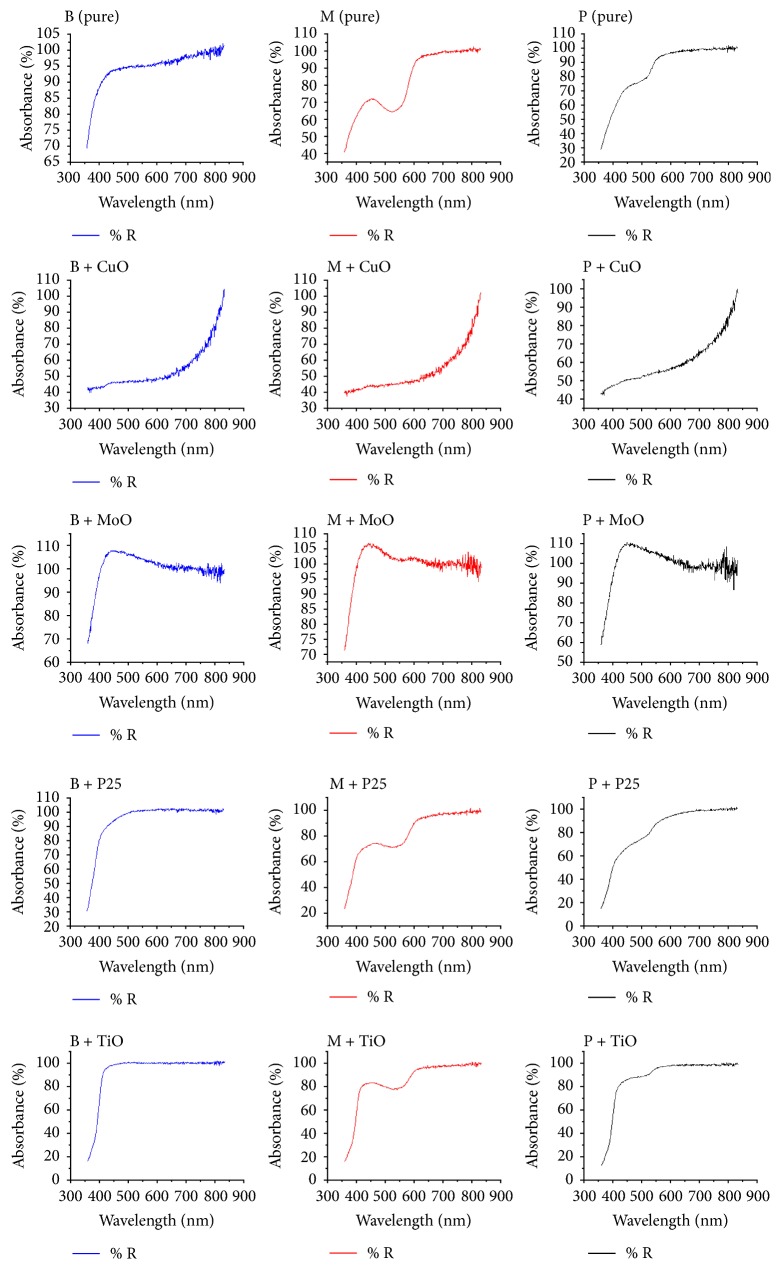
UV-Vis absorption spectra of samples in pure state and also doped with metal oxides nanoparticles.

Figure 4 shows the UV-Visible spectrograms of samples.

According to Figure 4, the peaks at 350 nm to 420 nm confirm the molecular vibration of oleo-gum-resin of olibanum [97]; the peak found near of 390 nm with another prominent peak at 480 nm confirms the molecular vibration of myrrh extract [98]; the peak found at 350 nm with vibration extending to another peak at 450 nm confirms molecular vibration of benzoin resin [99]; the intensity of spectra obtained at wavelengths 350 nm is typical for the crystalline structure of anatase + rutile (P25) and titanium dioxide (TiO_2_) [100]; the spectra showing intensity at wavelengths >300 nm typical for diametric and/or oligomeric species confirm the molecular vibration of MoO_3_ [101]. Lastly, distinct peaks observed at 600 nm with stretching band until 800 nm confirm the vibration Cu_2_O [102, 103].

**Figure 5:**
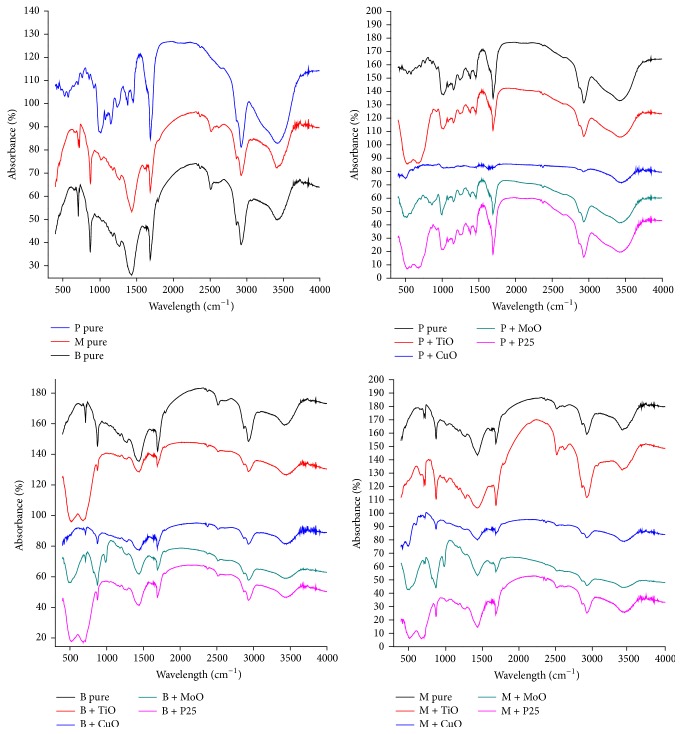
FTIR spectra of samples in pure state and also doped with metal oxides nanoparticles.

Figure 5 shows FTIR spectrograms.

According to Figure 5, the FTIR spectrum of olibanum shows a peak at 3422.37 cm^−1^ indicating the presence of –OH– group, and the peak at 1705.33 cm^−1^ indicates the presence of carbonyl group The stretching band at 3428 cm^−1^ (O–H) and at 2930 cm^−1^ (C–H) and the bending bands of C–H appear at 1455 and 1378 cm^−1^ and the stretching band of C=O in carboxyl group appears at 1717 cm^−1^, the stretching bands of C–O in carboxyl group are identified at 1243 cm^−1^, and the stretching band of C=O at 1737 cm^−1^ indicates the presence of esters in olibanum resin [90].

The FTIR spectrum of benzoin shows stretching band of carboxyl group (C=O) at 1719 cm^−1^, and aromatic skeletal bands at 1601, 1516, and 1451 cm^−1^, stretching band of C–O in carboxyl group at 1273 cm^−1^, and a bending band at 712 cm^−1^ show a phenyl group (Ph–H) peaks identified at 1207 to 1441 cm^−1^ and 1376 to 1450 cm^−1^ confirming the presence of coniferyl benzoate [104]; the peak found near to 1650 cm^−1^ evidences an aliphatic unsaturation with strong C=C bonds, the peak near to 1610 cm^−1^ shows weak aromatic unsaturation [105], the peaks found at around 2872 and 2923 cm^−1^ can be attributed to C–H asymmetric and symmetric stretching vibration of methylene [106], also the band observed at around 1450 cm^−1^ is due to C–H stretching vibration of methylene bridge, a peak at 1560 cm^−1^ can be assigned to stretching vibration of a carboxylate group (–COOH) [107], the broad peaks at 3500 cm^−1^ 3.420 cm^−1^ can be assigned to stretching of functional groups O–H [108], additionally peaks at 1580 to 1590 cm^−1^ correspond to stretching vibration of C=C–C aromatic rings [109], and lastly, a band observed at around 1400 cm^−1^ refers to C–H stretching vibration of vinyl [110] and broad peaks at 1070 cm^−1^ can be associated with stretching vibration of C–O–C [111].

The spectrum of myrrh shows the presence of broad bands located at 3.450, 1.630, and 1.550 cm^−1^ which are attributed to stretching of O–H, –COOH, and C=C and the shift of C=O vibration (symmetric stretching) of –COOH groups; an intense band at 1630 cm^−1^ confirms the molecular vibration of myrrh resin [105]; the stretching band identified at 1025 to 1200 cm^−1^ corresponds to the C–O stretching, while the weak bands at 1340 to 1450 cm^−1^ can be attributed to aliphatic hydrocarbons (CH_2_ and CH_3_) groups, groups of aldehydes (–CHO) and ketones (C=O), and the bending modes of bonds in alcohols (O–H), phenols (–OH), and carboxylic acids (–COOH); the bands at 1620 and 1650 cm^−1^ correspond to aromatic rings, while the bands around 2920 to 2930 cm^−1^ are analogous to the asymmetric stretching of the C–H bonds [106]; the strong broad band appearing at 3440 cm^−1^ can be assigned to the stretching vibrations of various groups in alcohols (O–H) and phenols (–OH) [105, 112].

The FTIR spectra of resins doped with titanium oxide nanoparticles reveal a small peak at 1640 cm^−1^ and a large broad peak between 3450 and 3200 cm^−1^, corresponding to the stretching vibrations of absorbed water, as well as hydroxyl (OH) groups present in the surface of TiO_2_–P25 nanopowder [113]; the broad peak between 600 and 400 cm^−1^ can be assigned to the presence of Ti–O–Ti bonds [114]. Concerning the copper I oxide addition, the peaks found at 529 and 602 cm^−1^ denotes the (Cu–O) stretching vibration of monoclinic CuO phase; the broad peak at about 490–620 cm^−1^ (central at 548 cm^−1^) was due to an overlap between Cu–O stretching vibration of Cu_2_O/CuO and (–OH) hydroxyl vibrations at 490–510 cm^−1^ [115]; the peak found at 620 cm^−1^ is related to Cu_2_O crystals [116], and the stretching bands found at 298 to 620 cm^−1^ match the crystalline structure of Cu_2_O [117, 118].

Regarding molybdenum nanopowder, two peaks found at 876.5 and 595.8 cm^−1^ are assigned to MoO_3_ phase [119]; the peak at 996 cm^−1^ was associated with the terminal stretching vibration of molybdenum in its oxidized form (MoO) that is an indication of layered MoO_3_ phase; the bands at 867 cm^−1^ and 558 cm^−1^ are assigned to stretching vibrations and bending vibrations of the Mo–O–Mo units [120]; the bands at around 3435 cm^−1^ and 1614 cm^−1^ can be attributed to the stretching and bending vibrations of (O–H) hydroxyl groups in the adsorbed water [121].

It is important to emphasize that FTIR absorption bands of different compounds in wood resin exudates may be discriminated according to their responses to a given thermal stimulation, because different compounds usually respond in different ways to the same stimulation. For example, the spectral bands of a volatile compound will decrease synchronously if this compound evaporates when the sample is heated [122]; since plant samples usually are complex mixtures, signal-resolving methods are necessary to find the spectral features of compounds of interest in the signal-overlapped IR spectra.

### 3.4. Differential Scanning Calorimetry (DSC)

**Figure 6:**
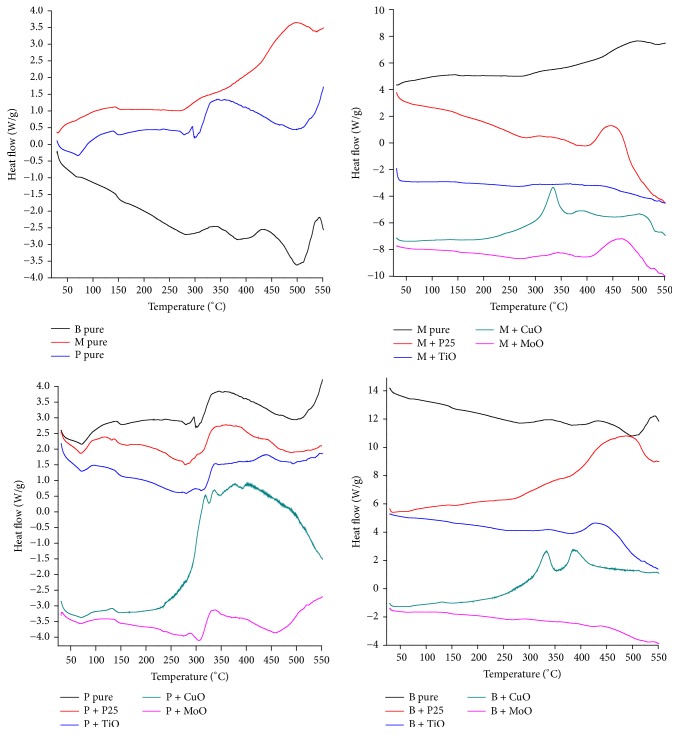
DSC phase diagram of samples in pure state and also doped with metal oxides nanoparticles.

Figure 6 shows the DSC curves of materials under analysis.

According to Figure 6, the thermophase diagram of myrrh in pure state presents characteristics of a semicrystalline polymer. The glass transition temperature (*T*_g_) of this resin occurs at 75°C; from ambient temperature to this point, the material presents glassy behavior; it is hard, inflexible, and brittle, from 75°C to 300°C and this resin exhibits rubbery behavior, at this range; it is soft and flexible, from 300°C to 500°C, and the material presents viscoelastic state; the resin reaches its melting temperature (*T*_m_) at 500°C. The peak at 150°C indicates a primary crystallization and the baseline change with a prominent peak at 300°C indicates a secondary crystallization, so the crystallization temperature (*T*_c_) of this material occurs between 150°C and 300°C. In comparison to other available polymers, this resin shows similar characteristics to commercial polyurea.

The resin of benzoin also presents characteristics of a semicrystalline polymer such as polyurea. From ambient temperature till reaching 80°C (*T*_g_), this biopolymers exhibits glassy state; from 80°C to 275°C, it presents rubbery state, with rigid crystalline phase and amorphous mobile phase; two exothermic peaks at 350°C and 420°C indicate that crystallizations occur at this temperature range (*T*_c_), from 275°C to 500°C; the resin presents viscoelastic state; finally, this resin reaches its melting temperature (*T*_m_) at 500°C.

Similarly, the resin of olibanum presents characteristics of semicrystalline polymers, such as polyester. Its glass transition temperature (*T*_g_) occurs at 80°C; from this point forward until 300°C, this biopolymer presents rubbery state; two peaks at 300°C and 400°C indicate that crystallizations occur at this temperature range (*T*_c_); lastly at 500°C, this biopolymer reaches its melting temperature (*T*_m_).

When the temperature of resins reaches its *T*_m_, the melting of crystallites occurs; at this point, the system power reaches the level needed to win the secondary intermolecular forces between the chains of the crystalline phase, destroying the regular packing structure, thereupon changing from rubbery state to viscous state. This transition only occurs in crystalline phase, so this interpretation only makes sense if it is applied to semicrystalline polymers [123].

It is evident that the addition of oxides nanoparticles will interfere in activation energy of particles by breaking existing chemical bonds between the atoms of each substance, thus favoring the occurrence of other chemical bonds and synthesis of a new substances, and also will influence the thermal-mechanical properties of samples (Ehrenstein et al., 2006) [124]. It will also depend on the degree of crystallinity, since higher crystallinity will result in a harder and more thermally stable but also more brittle material, whereas the amorphous regions provide certain elasticity and impact resistance [125, 126].

As exposed in the DSC signal, the addition of TiO_2_ and P25 reveals peaks at 300°C and 500°C; at high temperatures, TiO_2_ nanoparticles dehydrate and coarsen, the final stable phase upon grain growth being always rutile [127]. The overall process (phase transformation, water loss, and coarsening) is irreversible; the TiO_2_ and P25 samples contain adsorbed water which has the properties of bulk liquid water and a small fraction bound very tightly, probably in the form of hydroxyl groups [128]. The addition of Cu_2_O shows peaks at 300°C and 450°C, related to the removal of water from the surface [129] and the corrosion of copper nanoparticles [130], concerning the addition of MoO_3_. The occurrence of peaks at 300°C and 500°C is due to the removal of water and recrystallizations of phases present in the nanopowder [131].

In short, resins showed thermal behavior inherent to semicrystalline polymers such as polyester and polyurea; at some point, the molecules disposed in amorphous matrix obtain enough freedom of motion to spontaneously rearrange themselves into crystalline forms. This transition from amorphous solid to crystalline solid was evidenced by distinct exothermic peaks, as the temperature increases to 500°C samples, eventually reaching its melting point.

## 4. Conclusion

The biopolymers tested showed inherent characteristics of commercial available semicrystalline polymers; in most cases, the materials inhibited the proliferation of clinical pathogens under study, and, as expected, the addition of oxides nanoparticles increased the bacteriostatic effect. Although their addition was not well structured during the production of filaments and disks, nanoparticles remained disorderly dispersed over the matrix of resins, in most cases being encapsulated by the same.

## Figures and Tables

**Table 1:** Antibacterial activity of materials against selected pathogenic strains.

Oleo-gum-resin	Inhibition zone (mm)			
Nanooxide	*S. aureus*	*E. coli*	*P. aeruginosa*	*C. albicans*
M (pure)	1.5 ± 1.5	3.0 ± 0.4	5.5 ± 0.6	2.4 ± 3.2
P (pure)	2.0 ± 1.0	3.1 ± 0.5	4.7 ± 0.88	4.4 ± 2.4
B (pure)	1.1 ± 1.1	4.0 ± 0.4	4.0 ± 2.5	1.8 ± 1.8
M + TiO_2_	5.2 ± 0.18	8.8 ± 0.32	7.2 ± 0.28	6.8 ± 0.15
M + P25	6.0 ± 0.97	6.4 ± 0.11	6.1 ± 0.35	6.1 ± 0.26
M + MoO_3_	5.5 ± 0.22	7.0 ± 0.20	6.6 ± 0.32	6.7 ± 0.92
M + Cu_2_O	5.0 ± 0.28	6.5 ± 0.91	6.4 ± 1.57	5.5 ± 0.36
P + TiO_2_	5.5 ± 1.55	8.0 ± 0.14	7.7 ± 1.90	6.0 ± 0.21
P + P25	5.8 ± 0.10	5.2 ± 0.26	6.2 ± 0.88	6.0 ± 1.74
P + MoO_3_	5.5 ± 0.23	6.5 ± 0.36	7.0 ± 0.30	6.9 ± 1.59
P + Cu_2_O	5.6 ± 1.81	8.0 ± 0.53	7.4 ± 1.39	6.6 ± 2.13
B + TiO_2_	6.0 + 0.38	7.7 ± 0.77	5.0 ± 1.80	6.8 ± 1.27
B + P25	7.6 ± 0.53	6.2 ± 0.65	4.5 ± 0.36	6.1 ± 2.05
B + MoO_3_	6.1 ± 1.70	8.3 ± 0.40	5.2 ± 1.24	6.5 ± 2.46
B + Cu_2_O	7.5 ± 1.15	7.0 ± 0.88	4.8 ± 0.36	7.1 ± 2.15

*Note*. The experiments were done in quadruplicate and the results were interpreted in terms of standard deviation of mean diameter of zone of inhibition.
